# Effects of three personal resources interventions on employees’ burnout

**DOI:** 10.1038/s41598-023-49000-9

**Published:** 2023-12-06

**Authors:** Mariola Pérez-Marqués, Onintze Letona-Ibañez, Alejandro Amillano, María Carrasco, Silvia Martínez-Rodríguez

**Affiliations:** Faculty of Health Sciences, University of Deusto, 48007 Bilbao, Spain

**Keywords:** Human behaviour, Psychology

## Abstract

Personal resources are related to positive psychological states that can translate into lower burnout among employees. However, although these personal resources can be promoted through ad hoc interventions, there are few studies that analyze this type of interventions in workers. The aim of this study was to assess the effectiveness of three interventions on personal resources on reducing employees' burnout. To this end, it was hypothesized that a positive psychological capital intervention (PsyCap), a job crafting intervention and a combined intervention would have a positive impact on burnout levels. This research used a quasi-experimental, longitudinal, pretest–posttest design, with repeated measures and a waiting list control group. Study participants (N = 144) were all workers divided into three interventions and a control group. A noteworthy aspect of the study design is that the intervention was implemented as a voluntary online training activity. This study showed that personal resources interventions were effective in reducing burnout among employees. The PsyCap intervention and the combined intervention showed the greatest efficacy. Contrary to expectations, the combined intervention did not show significantly greater efficacy than the other two experimental groups. The study concludes with a discussion of its limitations and practical implications for future personal resources intervention studies.

## Introduction

The workplace has undergone continuous changes in recent years. The constant evolution of workers' demands and values, together with the relentless search for effectiveness and efficiency, pose a challenge to both workers and organizations^1^. The work setting is one of the environments that most affects people's physical and mental health. It also considerably influences their working life as a whole and some specific related aspects, including family life and interpersonal relationships^1^.

Burnout is a concept that is receiving increasingly more attention, due to its significant relationship with discomfort and with different factors in organizations such as staff turnover, interest and involvement in tasks, and extra-role behaviors^2^. This construct is defined as a syndrome of emotional exhaustion, feelings of low personal accomplishment, and depersonalization which arise as a response to chronic stress in jobs that require working with people^3^. The 11th revision of the International Classification of Diseases (ICD-11), published by the World Health Organization in 2019, categorized burnout syndrome as a problem associated with employment. According to ICD-11, burnout syndrome is a workplace phenomenon that is subject to interpretation and results from chronic work stress that has not been successfully managed^4^. Research has shown that burnout risk is associated with both an increased tendency to suffer from diabetes, depression, irritable bowel syndrome and hypertension and with higher healthcare spending by private insurers on employees categorized as being at high risk of developing burnout^5^.

This relationship between burnout and health impairment was captured by Bakker and Demerouti in their Job Demands-Resources (hereafter JD-R) model, which has served as a framework for numerous studies^6–8^. Over the past few years, research on the JD-R model has gradually increased and the model has become capable of predicting burnout. This theory comprises two basic elements, namely, job resources and job demands, and enables researchers to understand and predict the well-being and discomfort levels of working people; it is therefore mainly applied to the workplace^6,7,9^. Two distinct processes take place in the JD-R model: a health impairment process and a motivational process. The health impairment process is based on the fact that high demands lead to energy depletion and health problems due to the wear and tear of mental and physical resources, including burnout. Within the motivational process, lack of engagement due to scarce job resources results in unmotivated behavior associated with the development of burnout^6,8,10^.

Based on these data, different researchers have concluded that job demands predict factors related to job discomfort such as burnout, psychosomatic disorders, or intention to quit^10^. Job resources, for their part, are considered predictors of well-being, job satisfaction and commitment, since they cover autonomy and self-efficacy, among other basic human needs^7,8^.

Whereas in the JD-R model job demands and job resources are explained through different processes, both elements can interact and jointly affect workers' well-being. They can influence job performance in two possible ways. Firstly, job resources attenuate the impact that job demands have on indicators of distress such as burnout. In short, workers who have many resources at their disposal are better able to cope with their daily work demands^6,7^. Secondly, demands intensify the effect of job resources on motivation, whereby resources become more relevant and have a greater impact on engagement when demands are high^6–8^.

The initial focus of this model was on how job resources act as buffers against the impact of workers’ overload caused by the demands of the job. The importance of workers’ positive personal characteristics also became increasingly evident. At a later stage, the theory incorporated an approach that was not initially made explicit, which has become the starting point for numerous studies, namely, personal resources. It is based on the fact that the effects of resources and job demands on employees are different depending on their personal resources. These resources are the person's beliefs or self-assessment about the extent to which they can control and influence their environment^6,9^. They include cognitive and affective aspects of lower-order personality, such as self-esteem, self-efficacy, mastery, optimism, and hope, which motivate and facilitate goal achievement, even in the face of adversity and challenges^11^. These personal resources are malleable elements that can be cultivated through meaningful experiences or the learning and development of intervention programs^12^.

It has been shown that two variables defined as personal resources that are related to positive psychological states and work environment characteristics can translate into higher work engagement and lower burnout among employees: job crafting and positive psychological capital (hereafter PsyCap)^13^. Job crafting has been defined as the ability of the working person to modify their workplace tasks, work relationships, and attribution of meaning to pursue greater well-being. This personal resource makes it easier for workers to adapt to job demands and job resources and better match work demands. Within the JD-R model, job crafting is seen as a personal resource that buffers demands at work and allows workers to harmonize tasks and constraints by changing the balance between workplace demands and resources. This helps them to optimize their personal work goals and improves the work environment. In addition, workers who exhibit job crafting behavior manage to reduce the amount of demands in the workplace and avoid burnout^14^. Several studies have shown that job crafting is related to workers' well-being and to different general well-being indicators. Van den Heuvel et al.^11^ found that people who had this resource available were able to create a more motivating work environment and, therefore, improve their levels of emotional well-being. For their part, Slemp and Vella-Brodrick^15^ showed that job crafting was a predictor of the satisfaction of basic psychological needs (autonomy, skills) which, in turn, directly operated as predictors of subjective well-being (positive affect and life satisfaction) and independently of psychological well-being (a person’s optimal functioning). For these authors, in general, it follows that job crafting is an effective tool for improving workers’ well-being, both from an occupational and from a general point of view.

Van den Heuvel et al.^11^ pioneered the development of a job crafting intervention to teach participants how to perceive and modify the demands and resources of their job in order to adjust them to their needs and preferences. Several authors have shown that people with problems in their employment who change their work environment by better adapting it to their preferences and needs perceive lower levels of burnout, which are manifested as decreased exhaustion and disengagement. Increased job resources have also been related to lower fatigue and greater vigor at the end of the workday, thus preventing burnout^16^.

PsyCap is defined as a second-order factor that integrates four basic components: (a) self-efficacy, the confidence a person has in successfully achieving a challenging task; (b) hope, a person's perseverance in achieving their goals; (c) optimism, based on positive attributions about the present and the future; and (d) resilience, which entails overcoming challenges and adversities and growing personally as a result PsyCap is defined as a second-order factor that integrates four basic components: (a) self-efficacy, the confidence a person has in successfully achieving a challenging task; (b) hope, a person's perseverance in achieving their goals; (c) optimism, based on positive attributions about the present and the future; and (d) resilience, which entails overcoming challenges and adversities and growing personally as a result^17^. In turn, Avey et al.^18^ argued that PsyCap is composed of positive psychological resources that are related to well-being and positive organizational behaviors, such as job satisfaction and work-related outcomes. It is made up of four psychological variables which, when combined, give rise to a second-order construct that better predicts satisfaction and well-being than the items separately.

In turn, Avey et al.^18^ argued that PsyCap is composed of positive psychological resources that are related to well-being and positive organizational behaviors, such as job satisfaction and work-related outcomes. It is made up of four psychological variables which, when combined, give rise to a second-order construct that better predicts satisfaction and well-being than the items separately.

Luthans et al.^12^ proposed a psychological capital intervention (PCI) model to operationalize and apply PsyCap interventions. This model assumes that if the four components of PsyCap (hope, optimism, self-efficacy and resilience) are developed, they will overlap due to their interactive nature; the effects will then be greater than those that would be achieved by enhancing each component separately. Since then, numerous researchers have studied the effectiveness of this model and replicated it.

Along these lines, Jiang et al.^19^ reported that people with high levels of PsyCap present lower degrees of burnout and depression, and decreased anxiety symptoms. They found that job burnout was positively associated with depression and anxiety symptoms and negatively associated with PsyCap. Similarly, several studies have confirmed the negative association between PsyCap and burnout in various professions. They concluded that having these psychological resources enables individuals to manage stressful situations and cope with work demands without suffering from chronic stress or burnout^20^. Therefore, developing employees' PsyCap can encourage them to develop a positive psychological state that can prevent work-related distress^21^. A study conducted by Golmohammadian et al.^22^ revealed that PsyCap was related to less depersonalization and greater self-realization. Alipur et al.^21^ also found that the mean burnout score and its subscales (emotional exhaustion and depersonalization) significantly decreased in a group of 60 experts working at Iran Khodro Diesel Company after the implementation of a PsyCap intervention program.

These personal resources are characterized by their malleability; they can therefore be developed through interventions that have a positive impact on workers' burnout, including a growing ability to exercise control over their environment, cope with demands and achieve work goals. Personal resources thus facilitate personal development and the achievement of desired outcomes^23^. Bakker and de Vries^24^ argued that personal resources are involved in the self-regulation of burnout. In their view, employees with personal resources manage to prevent job burnout by using stable characteristics and skills such as emotional intelligence and a proactive personality, which in turn enables them to recognize and regulate their fatigue in a timely and effective manner to prevent burnout.

In conclusion, although personal resources can be promoted through ad hoc interventions, to our knowledge, there have not been many studies that analyze these types of interventions. In fact, no research has been conducted to compare the results that can be obtained by applying each type of intervention and the duration of the possible changes they bring about. For this reason, this study seeks to analyze and contrast the effectiveness of three interventions on personal resources aimed at reducing burnout. To this end, the following hypotheses are proposed:

### Hypothesis 1

Participants' levels of burnout will significantly improve after the PsyCap intervention, both compared to their levels before the intervention and compared to a control group.

### Hypothesis 2

Participants' levels of burnout will significantly improve after the job crafting intervention, both compared to their levels before the intervention and compared to a control group.

### Hypothesis 3

Participants' levels of burnout will significantly improve after the combined PsyCap and job crafting intervention (hereafter multicomponent), both compared to their levels before the intervention and compared to the interventions on an individual basis.

## Method

### Participants

The sample comprised 144 participants, of whom 101 participants were women (70%) and 43 were men (30%). The average age of the participants was 37 years (SD = 9.64). The sample exhibits educational diversity with 71.2% of participants having completed college, 4.5% having attained an elementary education level and the remaining 24.3% holding a secondary education diploma. Following the National Classification of Occupations provided by the Spanish National Institute of Statistics^4^, more than half of the participants had a highly qualified job (76.1%), while a smaller percentage of the sample were in less qualified positions (23.9%). Most of the sample members were employed (89.5%), mainly working full-time (76.3%) and with a permanent contract (56.3%). The multicomponent experimental group was comprised of 37 people, the PsyCap experimental group was made up of 34 participants, the job crafting experimental group had 32 members and the control group included 41 workers. No differences were found among the groups regarding age, gender, level of education, job position, and annual gross income, as depicted in Table 1. Additionally, no statistically significant differences were observed in any of the evaluated variables prior to the intervention, indicating a comparable baseline among the groups.

**Table 1:** Comparison of socio-demographic variables between intervention groups and control group.

	Exp group MC (1) n = 37		Exp group PC (2) n = 34		Exp group JC (3) n = 32		CTRL group (4) n = 41		t/χ^2^	*p*
	Mean/N	SD/%	Mean/N	SD/%	Mean/N	SD/%	Mean/N	SD/%		
Age	38.72	8.40	35.27	10.63	36.85	9.36	37.48	8.14	0.21	0.645
Gender										
Women	25	67.6	26	76.5	26	81.2	24	58.5	4.28	0.232
Men	12	32.4	8	23.5	6	18.7	17	41.5		
Educational level										
Primary education	0	0.00	0	0.0	2	6.3	5	12.2	3.96	0.646
Secondary education	9	24.32	7	20.59	14	43.75	9	21.95		
University studies	28	75.68	27	79.41	16	50	27	65.85		
Job position										
Management and leadership	0	0.0	1	2.9	0	0.0	2	4.9	7.10	0.503
Technicians and professionals	25	67.57	26	76.47	22	68.75	19	46.34		
Office employees	3	8.1	4	11.8	1	3.1	7	17.1		
Service workers	7	18.92	3	8.82	4	12.50	12	29.27		
Unskilled workers	2	5.41	0	0.00	5	15.63	1	2.44		
Annual gross income										
Less than 12,000	11	29.8	4	11.8	9	28.1	8	19.5	0.900	0.343
Between 12,000 and 24,000	6	16.2	17	50.0	20	62.5	23	56.1		
Between 24,000 and 36,000	2	5.4	12	35.3	3	9.4	8	19.5		
More than 36,000	18	48.6	1	2.9	0	0.0	1	2.4		

**Figure 1:**
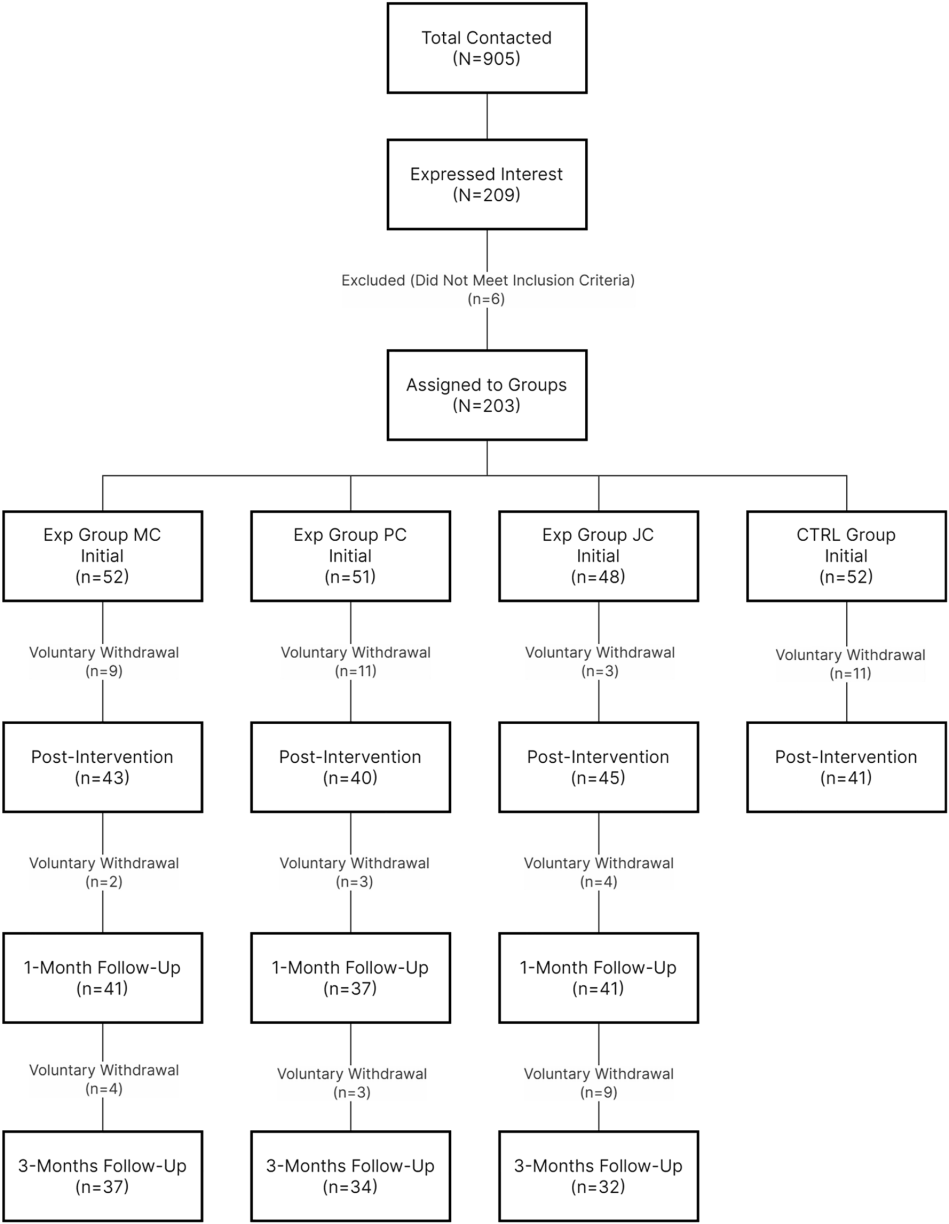
Flow diagram of study participant progression.

A detailed flow diagram illustrating the progression of participants through each stage of the study can be seen in Fig. 1.

### Procedure

The selection and recruitment of the sample collected for this research took place between January 2021 and May 2021. Participants, hailing from the health, education and social sectors within Spain, were non randomly contacted through LinkedIn and Facebook. Assignment to different groups considered age, gender, educational level, and professional status, with the aim of ensuring a balanced distribution and comparability among groups. In addition, specific inclusion and exclusion criteria were applied during participant selection. Inclusion criteria targeted individuals meeting the following requirements: current employment in a paid position, age of 18 or older, possession of a valid email address, and basic computer literacy skills. These criteria were established to ensure the enrollment of participants who not only met essential prerequisites but were also fully committed to the study.

Furthermore, exclusion criteria were implemented to uphold the quality and integrity of the collected data. Exclusion criteria included voluntary withdrawal from the study, incomplete participation in all intervention modules and incomplete responses to the required questionnaires. These criteria were enforced to maintain data integrity by excluding cases of participants who did not adequately complete all stages of the intervention and data collection.

In conjunction with this, the objectives, procedure and characteristics of the research were explained to the individuals who were interested. They were also informed that they would not be rewarded for their participation, except for a participant’s certificate. Those eligible individuals who decided to participate signed the informed consent form, which provided a specific description of the study, stated that participation was voluntary, and assured candidates that their data would be treated confidentially and would only be used for research purposes.

Each participant was non-randomly assigned to an experimental condition, adhering to a sequential enrollment strategy. The intervention programs were self-administered, of unequal duration and conducted online. It was decided to start with the longer intervention (multicomponent), followed by the intervention of medium duration (PsyCap) and ending with the shortest one (job crafting). Assignment to the waiting list control group was carried out simultaneously with that of the experimental groups. All participants completed an online questionnaire one week before the intervention’s implementation. To maintain the confidentiality of each participant, a four-digit code was used to represent sample members when completing the questionnaires. Once enrolled, they were sent a welcome email with login details (username and password), the link to the platform, and a brief help guide to access the platform. Participants could progress at their own pace, although it was recommended that they should complete one module per week to benefit from the course as much as possible. Participants were also contacted by e-mail on a weekly basis to assess whether the process was conducted appropriately, answer any questions from participants and guide them on their progress through the program.

To ensure intervention quality and integrity, a diverse range of strategies were integrated into the platform. These methods were crafted to actively engage participants and secure compliance with essential study prerequisites. Participants accessed the platform through unique username and password credentials, guaranteeing both identity verification and exclusive entry. Prior to progressing to the modules, participants were required to complete mandatory exercises, facilitating a structured and incremental learning process. Detailed activity logs meticulously tracked participant involvement, capturing access times, module durations, and exercise completion status. The implementation of automated email reminders played a significant role in sustaining participation and effectively addressing participant inquiries and concerns.

This study received ethical approval from the University of Deusto's Ethics Committee, Spain (Ref. ETK-23/17-18), and adhered to both national and international ethical guidelines, in line with the Declaration of Helsinki. Additionally, we confirm that all methods were performed in accordance with the applicable guidelines and regulations. Informed consent was obtained in writing from all participants, who were apprised of their right to withdraw at any time. Confidentiality and anonymity were assured by eliminating personal identifiers in all documents and materials related to the study.

This research used a quasi-experimental, longitudinal, pretest–posttest design, with repeated measures and a waiting list control group. The results were evaluated using pre- and post-intervention measures; in addition, the development of the variables over time was observed by using two follow-up measures (at 1 month and 3 months) in the experimental group.

### Methodology and contents

Today an increasing number of employees organize their free time in order to be able to engage in autonomous distance learning. Online self-learning can be an alternative to the face-to-face format, as it enables employees to organize their learning schedule over several days to suit their needs. These interventions consist of training tools with clear instructions, required materials, and post-tests to examine effectiveness, all of which provide step-by-step guidance for participants to achieve educational objectives independently^25^. Online self-administered positive psychology interventions (hereafter PPIs) have been shown to have crucial advantages over face-to-face programs, including speed, cost-effectiveness, and improved dissemination. To enhance these benefits, research conducted among the working population has pointed to guided, self-administered PPIs to successfully increase employee well-being^25^. However, not all online interventions are equally effective, as shown in the classification and regression tree analyses produced by van Genugten et al.^26^. According to these analyses, simpler, user-friendly interventions that require less time to understand are more effective, whereas those using normative comparison or identification of barriers and rewards for behavior change are less effective.

All these results support the use of online interventions in PsyCap and job crafting. Luthans et al.^27^ developed a two-session, 45-min web-based intervention that was effective in developing PsyCap levels. In addition, a PsyCap intervention consisting of four online training sessions aimed at improving each of the four PsyCap components was evaluated within five Chinese companies. The intervention was shown to be effective in significantly improving job performance, supporting the relationship between PsyCap and job performance^28^. The effectiveness of online job crafting interventions has also been studied by different researchers, albeit modestly. A study conducted by Verelst et al.^29^ assessed whether a job crafting intervention using the Job Crafting Exercise (JCE) elements proposed by van Wingerden et al.^13^ was able to increase the ability to modify relationships, task and task perceptions, and person-job fit. According to the results, the intervention effectively increased these variables, so it may be an interesting and accessible alternative to face-to-face job development interventions, especially for task-specific forms of development.

#### The PsyCap intervention in the study

This intervention consisted of five modules that participants could complete over a seven-week period. Table 2 shows the structure of each of the modules and their contents.

**Table 2:** Structure and content of the PsyCap intervention program.

Modules	Objectives
Module 1: Introduction	Learn about the concept of PsyCap Explore personal resources and their importance for well-being at work
Module 2: Developing self-efficacy	Develop self-efficacy based on the social cognitive theory developed by Bandura^30^
Module 3: Strengthening hope	Reinforce hope by following the primary cognitive processes defined by Snyder^31^
Module 4: Learning optimism	Develop optimism based on theories proposed by Seligman^32^
Module 5: Improving resilience	Improve resilience as indicated by Masten^33^

#### Job crafting intervention

This program consisted of four modules that participants could complete over a six-week period. Table 3 presents the structure of each module and its contents.

**Table 3:** Structure and content of the job crafting intervention program.

Modules	Objectives
Module 1: Introduction	Learn about the term “job crafting” Explore personal resources and their importance for well-being at work
Module 2: Fostering self-awareness	Learn about the concept of self-awareness Develop strategies to increase it
Module 3: Discovering job crafting	Learn the different forms of job crafting Identify the tasks, relationships and meaning of one's work
Module 4: Enhancing job crafting	Develop strategies to improve job crafting Establish actions to improve well-being at work

#### Multicomponent intervention

The multicomponent intervention consisted of nine modules that were completed over eleven weeks. This intervention began with the development of PsyCap and continued with the development of job crafting. The PsyCap components were intended to provide participants with greater resources that could be useful for job crafting.

At the end of the modules, participants completed an overall evaluation of the program, in which they assessed the effort made, the duration of the modules, and the perceived improvement of their personal resources after the program. Participants were also asked to provide some feedback, although the results were not part of the objectives of this research. The control group completed the same pre-treatment questionnaire as the intervention groups, as well as the post-treatment questionnaire after eleven weeks, as did the participants in the multicomponent intervention. Those in the control group who wished to do so could participate in the multicomponent program, which included the contents of the intervention in PsyCap and job crafting.

Regarding the structure of the interventions, each module included a theoretical section that provided evidence-based information and a practical section consisting of cases and exercises for reflection. Each module ended with a self-evaluation that requested their feedback on the concepts and a two-question questionnaire to rate the content of the session on a scale of 1 to 10.

### Instruments

The instrument used at the four measurement points in the four groups is described below.

#### Burnout

Burnout syndrome was measured using the Spanish translation of the Maslach Burnout Inventory-General Survey (hereafter MBI-GS) by Moreno-Jiménez et al.^2^. The MBI-GS was originally designed to assess burnout syndrome in health care professionals, focusing on the emotional exhaustion caused by continuous interactions with users or clients; however, it was later adapted to be applied to a more general work context, giving rise to the MBI-GS version^3^. This scale consists of 16 items distributed into 3 dimensions: emotional exhaustion (5 items), cynicism (5 items), and professional efficacy (6 items). A 7-point Likert-type scale was used to rate frequency, ranging from never (0 points) to every day (6 points). The Spanish adaptation of this scale has high reliability indices for the three dimensions (emotional exhaustion = 0.89, cynicism = 0.85, professional efficacy = 0.85), which are slightly higher than those of the original study (between 0.73 and 0.89). The levels of reliability found in this study were: emotional exhaustion = 0.90; cynicism = 0.90; and professional efficacy = 0.87.

#### Job crafting

To evaluate job crafting, the Job Crafting Questionnaire (JCQ) was employed, initially developed by Slemp and Vella-Brodrick^34^. The Spanish version of the JCQ was translated and adapted by Letona-Ibañez et al.^35^ to assess modifications in the work context, aligning with the theoretical framework proposed by Wrzeniewski and Dutton^36^.This scale has exhibited commendable reliability, with alpha coefficients of 0.88 and 0.86 for the total scale and 0.75, 0.86, and 0.79 for each of its three dimensions: task modification, cognitive modification, and relational modification, respectively. The JCQ consists of 15 items, which represent the three dimensions of the variable: task modification (5 items), cognitive modification (5 items), and relational modification (5 items). Items are evaluated using a 6-point Likert scale, ranging from 1 (almost never) to 6 (very often).

#### PsyCap

PsyCap was assessed utilizing the Spanish version of the Psychological Capital Questionnaire (PCQ) developed by Luthans et al.^17^. This questionnaire comprises 24 items, categorized into four factors: self-efficacy (6 items), hope (6 items), optimism (6 items), and resilience (6 items). A 6-point Likert response scale was employed, providing options ranging from 1 ("Strongly Disagree") to 6 ("Strongly Agree") to determine each participant's level of agreement with each statement. Regarding the reliability of the scales, adequate composite reliability indices were obtained for all dimensions of the PCQ: 0.87 for self-efficacy, 0.86 for hope, 0.81 for resilience, and 0.86 for optimism.

### Data analysis

An analysis of variance was conducted to analyze the results using the selected design (pre-, post-, and double follow-up test contrast for three groups). This involved calculations within subjects, between subjects and interaction effects using an *F*-test and *p* values. The assumption of sphericity in the variance–covariance matrix was checked using Mauchly’s test of sphericity and Levene’s test was employed to check homoscedasticity. To estimate effect sizes, eta squared (η^2^) and their equivalent Cohen’s d were calculated. In order to estimate pre-post change, difference scores (M_dif_) were computed (post-test values minus pre-test values), with positive values indicating an increase in the variable and negative ones indicating a decrease. To analyze differences in these scores, an analysis of variance was done (*F*-test) using the Scheffé test and Hedge’s g index to estimate effect size.

Utilizing G*Power v.3.1.9.7, the sample size was determined by setting a target power of 0.80 and applying a moderate effect size of d = 0.30, chosen to detect meaningful, realistic effects aligned with the study objectives. A two-tailed significance level of 0.05 was employed to support the robustness of the findings.

### Ethics declarations

All procedures performed in studies involving human participants were in accordance with the ethical standards of The Research Ethics Committee of the University of Deusto (Ref. ETK-23/17-18).

### Informed consent

Informed consent was obtained from all individual participants included in the study.

## Results

Preliminary analyses showed that the assumptions of sphericity in the variance–covariance matrix were adequate and there was good homoscedasticity.

**Figure 2:**
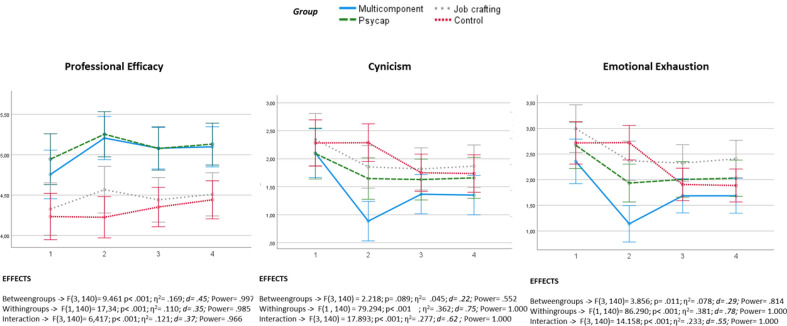
Burnout indicator outcomes across groups.

Figure 2 shows how the scores of the three groups fared in the three burnout dimensions. Statistically significant interaction effects were found for all scores, with relevant effect sizes (Cohen’s d values ranging between 0.37 and 0.62), indicating a differential response between the groups. While an improvement in burnout measures (a decrease in cynicism and emotional exhaustion, an increase in professional efficacy) was observed in the three intervention groups (job crafting, PsyCap, multicomponent), this trend was not seen in the control group.

In order to assess pre-post change differences between groups, the mean differences of each group were calculated and compared with each of the other groups. Table 4 shows that the test was significant and the post-hoc tests revealed that there were differences when each intervention group was compared to the control group. As can be seen in Table 4, multicomponent and PsyCap intervention groups (groups 1 and 2) showed statistically significant differences in relation to the control group for the three dimensions studied. They also exhibited a notable effect size (Hedge’s g > 0.80). However, the job crafting intervention group had statistically significant differences with respect to the control group, and effect size was irrelevant (Hedges’s g < 0.1). Differences between the three intervention groups were not significant except for emotional exhaustion and professional efficacy; when the job crafting group was compared with multicomponent and PsyCap groups, respectively, a significantly greater improvement was seen in the latter two.

**Table 4:** Between-group contrast of change scores (Mdif; Pre-Post on burnout dimensions: cynicism, emotional exhaustion, and professional efficacy).

	Exp group MC (1) n = 37		Exp group PC (2) n = 34		Exp group JC (3) n = 32		CTRL group (4) n = 41		Statistics test		Post-hoc test, effect size (g)					
	M_Dif_	SD_Dif_	M_Dif_	SD_Dif_	M_Dif_	SD_Dif_	M_Dif_	SD_Dif_	F	*p*	1 versus 2	1 versus 3	1 versus 4	2 versus 3	2 versus 4	3 versus 4
Cynicism	− 1.22	1.16	− 0.45	0.48	− 0.49	0.69	0.00	0.03	19.31	< 0.001	0.84**	0.74**	1.51**	0.07	1.37	1.06*
Emotional Exhaustion	− 1.22	1.28	− 0.73	0.91	− 0.62	0.74	0.00	0.17	13.13	< 0.001	0.42	0.55	1.35**	0.13	1.17**	1.23*
Professional Efficacy	0.45	0.91	0.31	0.28	0.24	0.40	− 0.01	0.06	5.39	0.002	0.20	0.29	0.72**	0.20	1.59	0.91

*Statistically significant at *p* < 0.05.

**Statistically significant at *p* < 0.01.

**Figure 3:**
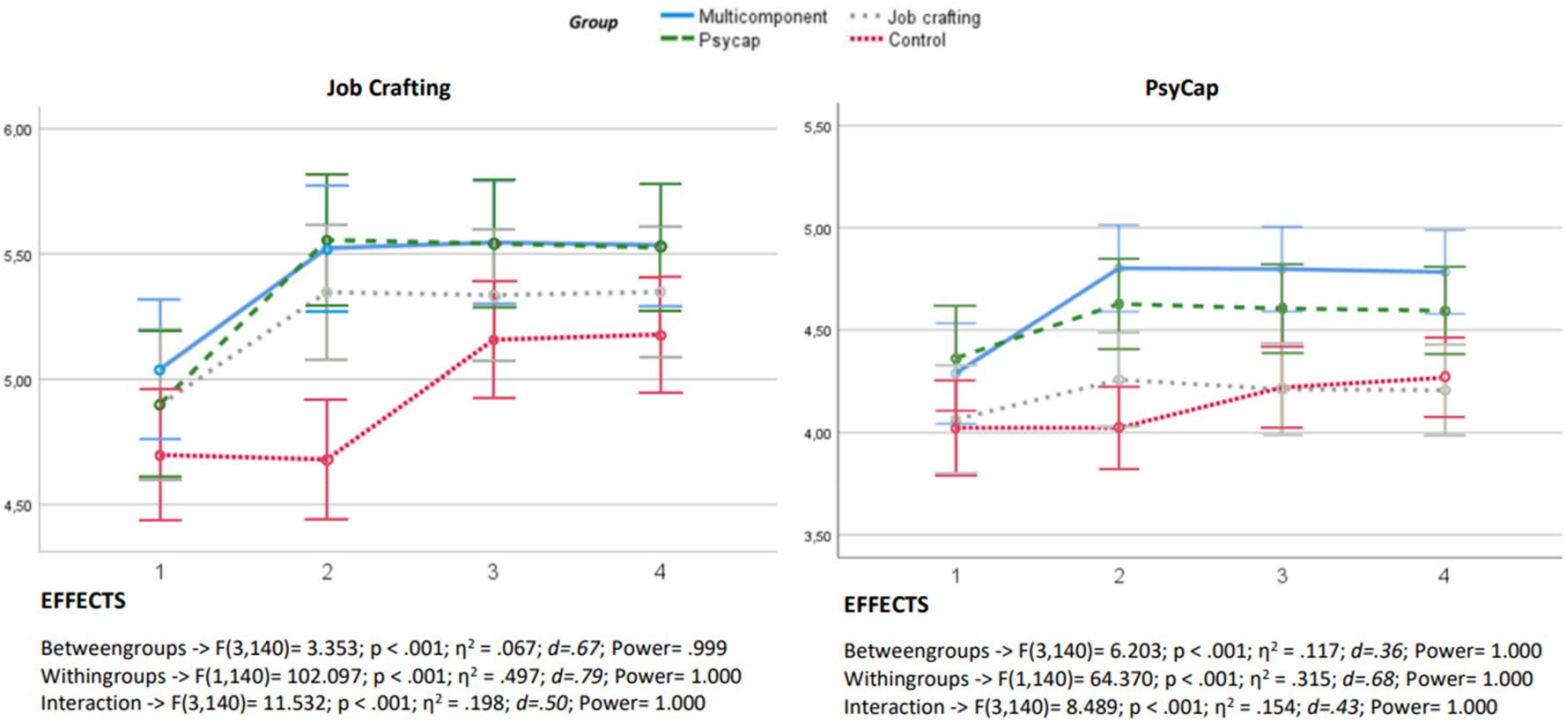
PsyCap and job crafting indicator outcomes across groups.

Figure 3 shows the temporal trajectories of scores across four groups, focusing on two intervention target variables (job crafting and PsyCap). A distinction was revealed both intergroup and longitudinally, implying disparities in mean scores among distinct groups and intra-group fluctuations across temporal measurements, thereby elucidating the heterogeneity in treatment responses and group-time interactions. Notably, the interventions, encompassing both job crafting and PsyCap, not only precipitated a positive inflection in scores through the temporal span but also sculpted divergent pathways of evolution among the participants. This differential impact is supported by moderate to large effect sizes and a statistical power ranging between 0.999 and 1.000. Despite the evident improvement in job crafting and PsyCap metrics among the three interventional groups (job crafting, PsyCap, multicomponent), this trajectory was not observed in the control group.

## Discussion

This study shows that interventions based on self-applied personal resources can be effective in reducing burnout among employees. Three different interventions aimed at reducing burnout were used; one of them was based on job crafting, another one on PsyCap, and the third one combined both personal resources. The interventions relied on the JD-R model^7–9^ which establishes a mutual relationship between personal and work resources in reducing burnout levels.

In the intervention on PsyCap, it was observed that the levels of the three components of burnout improved significantly with respect to the pre-intervention level. While there was a slight trend toward nonsignificant recovery in the follow-up measures in the variables of cynicism and emotional exhaustion, this contrasted with the trend toward improvement seen in the professional efficacy measures. However, these results were not replicated in the differences with respect to the control group in the variables cynicism and emotional exhaustion, where no significant differences were found in the follow-up measures due to the decrease in follow-up measures in the control group.

These results were replicated almost in their entirety in the three variables of the job crafting intervention, the only discrepancy being a nonsignificant difference in the follow-up measures of professional efficacy with respect to the control group. In view of these results, it can be concluded that the data partially support the first two hypotheses that participants' burnout levels would significantly improve after the PsyCap (Hypothesis 1) and job crafting (Hypothesis 2) interventions, both compared to their level before the intervention and compared to the control group.

The results in the multicomponent intervention showed significant improvement in all three variables with respect to pre-intervention measures. In cynicism and emotional exhaustion, nonsignificant recovery was observed with respect to the pre-intervention measures at follow-up. In professional efficacy, improvement remained stable in the follow-up measures. However, despite obtaining a significantly greater improvement than the previous interventions in the cynicism and emotional exhaustion variables, these results were not replicated in professional efficacy, which were similar to those in the PsyCap intervention. Therefore, Hypothesis 3 was partially supported by the data, as the burnout levels of the participants significantly improved after the multicomponent intervention compared to their pre-intervention levels and to the separate interventions, with the exception of professional efficacy.

The results showed that cynicism and emotional exhaustion can be reduced by means of interventions based on personal resources. A decrease in the values of the cynicism and emotional exhaustion variables was seen in all three groups. Several authors have indicated that cynicism has been rarely studied in the literature due to the high persistence and low tolerance for change associated with it^10,37^. A PsyCap intervention conducted by Stratman and Youssef-Morgan^38^ was found to be effective in reducing cynicism, supporting the idea that positive resources and their components can counteract the cynicism that contributes to increased burnout. The interventions in the study resulted in decreased cynicism. However, it cannot be forgotten that the outcomes of interventions can be influenced by other elements pertaining to work. Specifically, Gong^39^ stated that there are five fundamental factors involved in work well-being, namely, the job itself, promotion, salary, managers, and co-workers. In other words, along with individual factors, organizational factors are also very important in their determination. In the light of these results, it would be appropriate to delve deeper into the influence of these elements on the burnout of employees.

It is worth noting the worsening of the levels of emotional exhaustion and cynicism in the multicomponent group in the follow-up measures, although they did not reach pre-intervention levels. Some studies that examined the wide variety of occupational health and wellness interventions agreed that multicomponent interventions tend to have a greater effect than single interventions; however, they emphasized the absence of research on how to combine interventions for best results^40,41^. This lack of research on the correct combination of interventions may explain the inconsistency of the results with previous literature. Therefore, it would be worth examining whether using a different combination of job crafting and PsyCap interventions, or giving different weights to the intervention components could lead to greater stability of results.

While the post-treatment values of cynicism and emotional exhaustion in the experimental groups in job crafting and PsyCap remained stable, this was not the case in the control group, in which a large decrease could be seen that made the differences with respect to the groups nonsignificant. It is interesting to note the improvement in the values of cynicism and emotional exhaustion in the control group. It is worth mentioning that the sample was mainly composed of teaching professionals and that the follow-up measurements were taken in July and September, coinciding with the summer vacation and the beginning of the school year, respectively. Whereas it is true that vacations contribute significantly to the well-being of workers, their beneficial effects fade approximately two weeks after resuming work, a phenomenon known as the fade-out effect^42^. Research on this topic is scarce. Consistently with our results, Reizer and Mey-Raz^43^ found that the levels of emotional exhaustion decreased significantly in a sample of Israeli employees immediately after vacation and two weeks after returning from vacation, although the beneficial effects of taking a break from work tended to fade over time. Furthermore, these authors added that organizational factors such as perceived organizational support maintained the decrease in employee burnout, while perceived job insecurity increased employee burnout. That is why carrying out a larger longitudinal study would be useful to observe the results in subsequent follow-ups and check whether the levels in the control group persist or return to pre-vacation levels.

The results showed that personal achievement can be increased through interventions based on PsyCap and combined interventions, with the exception of job crafting. The improvement was maintained in post-treatment measures. In line with the results, previous studies found a strong relationship between PsyCap and professional efficacy^44^. The PsyCap intervention proved to be effective in increasing self-efficacy, one of the factors that make up this construct. As Ozer^45^ stated, the perception of self-efficacy together with outcome expectations will determine one's decisions and actions. These results were based on Bandura's^46^ social cognitive theory, which showed how self-efficacy has strong impact on human behavior linked to motivation and perseverance, and consequently can be related to professional efficacy. Other authors have agreed that intervention in PsyCap produces an increase in professional effectiveness^47^. However, Luo et al.^48^ argued that, in addition to personal resources, if the work environment does not have organizational resources, the increase in professional effectiveness may be difficult to achieve. Job efficacy can be enhanced by establishing supportive relationships, which can yield beneficial outcomes by improving self-efficacy, fostering coping mechanisms, and experiencing fewer negative and more positive emotions^49^. The present research aimed to increase professional efficacy using a job crafting intervention by means of its components. These results were not congruent with what was expected from the review conducted. According to the authors mentioned above, there is a need to increase self-efficacy values for the supportive relationship to be effective, which suggests that, in order to improve these values, positive psychological development needs to precede relational crafting.

Finally, the multicomponent intervention program provided better results that led to a lower level of emotional exhaustion and cynicism and a higher level of personal efficacy compared to the control group and the other experimental groups. This is consistent with the research conducted by different authors which argued that multicomponent interventions are more beneficial than those with a single component. Previous research has reported higher effect sizes for multicomponent interventions than those found in studies that examined the individual efficacy of the strategies included in the intervention, pointing to them having a joint effect^50^. The larger effect size found in this study suggests a combined effect of the different strategies incorporated into the intervention. Focusing on personal resources interventions, the literature review on combined job crafting and PsyCap interventions revealed that these are limited. Van Wingerden et al.^13^ argued that a combined personal resource intervention (based on PsyCap and job crafting) will be more effective than each of the interventions separately, as workers' enhanced personal resources will contribute to improving their personal confidence and job crafting levels^23^. Considering the scarcity of professionals' time, in order to maximize the effectiveness of interventions for workers, it is advisable to assess the effectiveness of combined interventions rather than using a single intervention. Therefore, interventions that combine different constructs could be especially useful for the reduction of burnout among workers.

In summary, the multicomponent and PsyCap intervention groups showed the greatest efficacy out of the three dimensions studied. Contrary to expectations, the multicomponent intervention only showed significantly greater efficacy than the other two experimental groups in the cynicism variable. Professional efficacy showed greater improvement and stability over time.

### Limitations and future research

The limitations of this study should be recognized. The research used convenience sampling, which is subject to multiple biases in external statistical validity. Therefore, results can only be applied to the group of participants in the study and cannot be generalized to the target population. In addition, the participants in the intervention group were mostly female, highly educated, and came from health care or educational settings. This gender imbalance in the study is a consequence of the significantly higher number of women in health care and educational positions. Future studies could increase the number of male participants and of sample members with lower levels of education in order to better represent the general population. The job crafting intervention showed the worst results, which could be attributed to the short duration of the intervention. This intervention was based on the Michigan Job Crafting Exercise developed by Wrzesniewski et al.^14^, a brief exercise that addresses three components: relational, task, and cognitive. Longer interventions for the different components could therefore have led to more significant results. Different authors have considered that a brief intervention is one that has a maximum duration of about five hours, approximately the time needed to complete the intervention program. However, research is scarce and there is no strong evidence or consensus indicating the optimal duration of interventions^41^. An investigation to further develop this issue would be appropriate to optimize organizational concerns.

Whereas it is true that there are multiple self-applied online treatment programs, access to them is not equally affordable for all; in addition, there may be problems for some people regarding the use of new technologies depending on age and educational level. The use of these tools should be therefore incorporated into the daily life of the general population in order to generalize these intervention programs. Although the dropout rate was not high in this study, it was higher than expected in a face-to-face intervention. To solve this problem, the motivational aspects of the intervention could be addressed through qualitative evaluations of the intervention programs. The effectiveness of the interventions has been evaluated in its entirety, so a possible avenue of research for the future could involve conducting an individual evaluation of each one of the program modules in order to analyze their contribution to the reduction of burnout and the development of a more effective combined intervention program.

## Conclusions and practical implications

In practice, this study shows the importance that interventions on personal resources can have in reducing burnout, which translates into improved workers' well-being. It also indicates that organizations should play a role in investing in these interventions and incorporating certain organizational changes to promote the well-being of their workers, allowing them to apply the strategies acquired during training. To this end, it is important that organizations pay more individualized attention to the needs of each employee based on each of their resources, motivations, and difficulties. This would entail analyzing whether the demands of the job are adequately suited to the employee's profile. However, for these changes to be effective, it is essential that managers are aware of the benefits that these interventions can have in terms of occupational health and motivation. Awareness-raising through training campaigns could be useful for this purpose.

In conclusion, this study shows that personal resources interventions can be effective in reducing burnout levels and have a positive influence on workers' well-being. Although the results seem promising, some contrast with the hypotheses formulated. As this is a recent avenue of research, further studies are needed to better understand the factors that make these interventions more effective, as well as the most appropriate conditions for their application.

## Data Availability

The datasets generated during and/or analyzed during the current study are available from the corresponding author on reasonable request.
